# Preclinical model for lumbar interbody fusion in small ruminants: Rationale and guideline

**DOI:** 10.1016/j.jot.2022.10.006

**Published:** 2022-11-15

**Authors:** Anneli Duits, Daniela Salvatori, Jacobine Schouten, Paul van Urk, Steven van Gaalen, Karsten Ottink, Cuhmur Öner, Moyo Kruyt

**Affiliations:** a University Medical Center Utrecht, Department of Orthopedic Surgery, Utrecht, the Netherlands; b Utrecht University, Faculty of Veterinary Medicine, Anatomy & Physiology, Department of Clinical Sciences, Utrecht, the Netherlands; c Acibadem Internal Medical Center, Department of Orthopedic Surgery, Amsterdam, the Netherlands; d University of Twente, Department of Developmental BioEngineering, Enschede, the Netherlands

**Keywords:** Lumbosacral region, Models, Animal, Goats, Sheep, Spinal fusion, Translational research

## Abstract

Lumbar interbody fusion (IF) is a common procedure to obtain fusion of the spine by replacement of the intervertebral disc with a cage. Optimization of spinal cages is ongoing to reduce complications such as a pseudoarthrosis and subsidence of the cage. IF animal models (primate, dog, pig, goat and sheep) remain important to assess implant effectivity. But currently the available literature is dispersed and not IF model specific. Therefore unwanted inconsistencies between studies occur that limit generalizability. Based on our experience, anatomical preparation and literature research, we present a rationale for species selection and a practical guide for the surgical procedure in the goat animal model.

**The translational Potential of this Article:**

Rigorous methodologic design is an important means to improve translational value and generalizability of large animal IF efficacy studies. This paper provides a rationale and practical guide for animal selection and surgical decision making that can help reduce unnecessary variation between models and improve methodologic rigor and documentation for future experiments.

## 1 Introduction

Spinal fusion is a common treatment for a plethora of spinal diseases with incidences showing an upward trend worldwide [1]. Nowadays, interbody fusion (IF) is performed in the majority of cases, either or not combined with posterolateral fusion (PLF) [2]. To facilitate IF, in 1988 cages were introduced in order to provide a wide range of design possibilities with the final aim to optimize its biomechanical and osteogenic properties [3]. These design aspects are of interest for many scientific fields and relevant preclinical models are required [4,5].

Large animal models are used to demonstrate proof of concept and predict clinical efficacy. The reliability and translational value of results obtained in animal models are very much dependent on the rigor of the experimental design [6]. However, literature about model selection and surgical technique is dispersed and often not specific for IF models. We were confronted with this shortcoming while preparing a preclinical lumbar IF study according to Good Laboratory Practice (GLP) rules [7].

The PREPARE and ARRIVE guidelines support best practices in the design, conduct and reporting of animal studies and therefore are important from the experimental design process until publication [8,9]. The purpose of the research influences the design of the study, but there are currently no well-defined criteria and guidelines to justify the model choice of the animal model [10]. Animal selection is based on in-depth knowledge of the researcher, taking into consideration the validity of the model to the human situation. Previous studies have reviewed spinal morphometry [11,12], bone characteristics [[13], [14], [15], [16]] and spinal biomechanics [17], but never specifically to guide animal selection for IF animal models. Small ruminant anatomy descriptions are available both from anatomical atlas and technical papers [18,19], but descriptions are often brief and lack the detail to guide surgical decision making.

In this paper we aim to provide a rationale for animal selection and a practical guide for IF procedures in small ruminant IF models. In the first part of this review a synthesis of key literature is provided for the selection of IF animal models. The second part of the review focusses on surgical decision making in terms of level selection, endplate-preparation and spinal stabilization strategies. In-depth knowledge about small ruminant anatomy relevant for IF surgery is provided, based on anatomical preparation of a goat and review of the literature. It is shown how spinal anatomy in small ruminants is different from that in humans and how this affects the surgical procedure. The variation in current model use is discussed, based on a systematic review of small ruminant IF literature and a guide for the surgical procedure is provided based on relevant surgical anatomy and our experiences with a goat lumbar IF model.

## 2 Materials & methods

### 2.1 Literature review

A narrative review was conducted for section ‘1. Selection of animal model’ and section ‘2.1 relevant surgical anatomy’. Literature was identified using Pubmed and Google scholar.

For sections 2.2, 2.3. a systematic literature search was performed, including papers written in English from 1985 to September 2021 describing a lumbar IF model in sheep and goats. For a detailed description of the search strategy and paper selection see supplement A.

### 2.2 Anatomical preparation

In order to study relevant surgical anatomy, anatomical preparation of a female Dutch milk goat (weight 51 ​kg, age 4 years) was performed, supplementary to the findings in literature for section 2.1. To reduce animal use, the animal was taken over after termination from a non-related animal study in which spinal anatomy was preserved and was directly embalmed after planned euthanasia. Latex (Pliatex) stained red (arteries) and blue (veins) was injected in the aorta and vena cava respectively to fixate the arteries and veins. The spine and iliac crest were obtained preserving the relevant anatomy. The right psoas major, psoas minor and iliac muscles (IM) were carefully extracted to visualize the spinal vasculature. The left psoas major, psoas minor, IM and the lumbar plexus were preserved.

### 2.3 Experience with a goat lumbar IF model

We used our experience with a cage efficacy trial in 20 skeletally mature Dutch milk goats to write sections ‘2. Planning the IF surgical procedure in small ruminants’ and ‘3. Pitfalls and considerations regarding caprine anatomy’. All procedures were performed in accordance with Dutch legislation for animal research and were approved by the national animal ethics committee (“centrale commissie dierproeven (CCD)” (protocol number AVD115020172985)). In all goats the L2-3 and L4-5 received an IF device that was stabilized using a L2 to L5 screw and rod instrumentation construct. For a detailed description of housing, husbandry, animal care and monitoring see supplement B [8].

## 3 Results

### 3.1 Selection of IF large animal model

Primate, dog (canine), pig (porcine), goat (caprine) and sheep (ovine) are well known large animal models. As a general rule a model should resemble the clinical situation as much as possible. A rational approach for selection of an IF large animal model should be based on spinal morphometry, bone characteristics and spinal function. These factors are weighted against practical issues like availability, acceptance and costs to guide final selection in terms of animal species, genetic aspects, sex and age [20].

### 3.2 Lumbar spine morphology

The surface area of the endplate and the intervertebral disc (IVD) height determine implant size and are summarized in Table 1. The vertebral size and shape of animals differs from that in humans. The animal endplate dimensions are smaller, while vertebral height is taller and the IVD height less, generating more slender vertebrae [21].

**Table 1:** Numbar morphometry reported in different studies.

Parameter	Human	Pig	Baboon	Sheep	Goat
**IVD height (mm)**	11.07 ​± ​2.31	5.12 ​± ​0.96	5.32 ​± ​2.44	3.80 ​± ​0.42	4.09 ​± ​0.68
**Vertebral body width (mm)**	49.01 ​± ​4.62	37.57 ​± ​2.85	36.23 ​± ​6.04	27.03 ​± ​2.19	24.30 ​± ​2.02
**Vertebral body depth (mm)**	36.36 ​± ​3.34	25.28 ​± ​2.04	22.69 ​± ​1.46	19.18 ​± ​1.43	18 ​± ​1.65

Values are presented as a mean ​± ​standard deviation. The presented values are weighted means and pooled standard deviations based on the literature. Human values are based on [11,[22], [23], [24], [25]], porcine values from Refs. [11,22,26], primate values from [11,23], sheep values from Refs. [11,23,[26], [27], [28]], goat values from Ref. [29] and our own microCT analysis of 18 caprine spinal segments.

### 3.3 Bone characteristics

On a macroscopic level, the lumbar vertebrae consist of a cortical lining, including the endplates, and a trabecular core in both humans and animals, but differences in bone microstructure exist.

Cortical bone in humans largely consists of well-organized secondary bone, that is the result of coordinated bone remodeling [13]. Only mature non human primates (NHP) show a similar bone structure. In other large animals, remodeling is minimal until adolescence and the primary plexiform bone type dominates [30]. Only long after maturation there is a transit to secondary bone which is age related per species [13,14].

Differences in endplate characteristics, and how this influences IF, are less extensively studied. In humans, the endplate consists of the cartilaginous endplate (CEP) and the subchondral bone plate or bony endplate(BEP). Before skeletal maturity a growth zone is present between the CEP and trabecular bone, that later on is replaced with the BEP [31]. With IF procedures the cartilage is removed and the cage is positioned directly in between the bleeding bone of the adjacent vertebrae. Endplate characteristics have been studied and compared for goats, cows, dogs and NHP [32,33]. The NHP endplate is very comparable to that in humans, except that the growth plate remains after skeletal maturity, and therefore no clearly defined BEP is present [33,34]. In cows, goats and dogs the growth plate also persists and is located below a vertebral epiphysis. The vertebral epiphysis contains the BEP which is thicker compared to that in humans, but decreases as the species size increases [35]. Incomplete CEP removal negatively influences fusion and results in intervening cartilaginous tissues [36,37]. Therefore careful removal of the CEP is important in both human and animal surgery. Whether the growth plate in animals remains active and how this affects the outcome of IF models is unknown [38].

The vertebral trabecular bone consists of well-structured lamellar bone in both the human and animal mature skeleton. The trabecular bone differs for trabecular strut thickness and density of osteocytes among species. Trabecular strut thickness increases with species size whereas trabecular strut number and osteocyte density decreases [39]. Therefore, the larger the species, the more comparable the animal trabecular bone is to that in humans.

Although NHP bone characteristics are considered most closely related to humans, high interspecies variability and biologic responses that were not predictive for humans exist [14]. The characteristics of skeletally mature dogs, goats, sheep and pigs actually differ only moderately from humans and therefore none of these models is clearly superior in terms of bone characteristics [14].

### 3.4 Spinal function

The applicability of quadrupeds for spinal research was long debated for the obvious postural differences, but the quadruped spine resembles human spinal kinematics relatively well and the NHP spine does not perform any better in comparison to quadrupeds [40]. The spinal function is similar to humans as the column supports the body with a substantial range of motion (ROM) that is stabilized by muscle forces [41,42]. Humans and quadrupeds show the same trabecular bone architecture consistent with axial loading. However it's likely the quadruped vertebrae are subject to higher stresses as the BMD is higher and the endplate surface is smaller [17].

### 3.5 Practical considerations

Despite growing ethical and societal concerns, currently no *in vitro* nor computer model can replace the IF animal model. And, as fusion assessment with current radiographic techniques is still suboptimal [43], animal models remain important to assess cage efficacy and improve understanding of spinal fusion. Use of NHP is restricted due to ethical concerns related to the behavioral and cognitive resemblance to humans [44,45]. In addition, it demands extensive welfare requirements that are only available in specialized facilities. Currently there is no consensus nor a compelling need for this model, therefore other IF models are preferred.

Consequently, preclinical animal research is mostly performed with farm animals and pets [46]. The spinal dimensions of the bovine species (cow) better approximate those in humans, but no anesthetic procedures nor theater equipment exist for such a large animal [24]. Skeletally mature dogs, goats, sheep and pigs are equally suitable [14] and a shift towards pigs, goats and sheep is seen [13,47]. Despite the general assumption of better resemblance to humans, the skeletally mature pig, that can weigh up to 350 ​kg, is practically difficult to handle [15]. Therefore small ruminants are currently the most practical animal model as availability is good, the price is relatively low and animal handling is straight forward.

### 3.6 Animal species, genetic aspects, sex and age

Sheep are more frequently used for IF models [47], maybe due to the more docile behavior compared to the more curious nature of goats [46]. Based on our experience with goats, we don't recognize this as a drawback and because the goat interacts more with the personnel, discomfort is easier to detect. For sheep, age dependent variations of BMD are described, making sheep between the age of 7 and 9 years most comparable to humans [15,16]. BMD variation is not described for goats, but similar bone formation to humans was already shown for much younger ages [48]. Genetic variation of small ruminants is inevitable, as no specific breeding for research purposes is available. However, dairy farm animals are relatively homogeneous and can be used after their milk production has ceased, which limits the costs and is ethically favorable. For this reason mostly females are used.

### 3.7 Planning the IF surgical procedure in small ruminants

After selection of the animal model the following five items are important:

1) Excellent knowledge of animal anatomy.
2) Selection of the lumbar levels, taking into account anatomical constraints and the 3R concept (replacement, reduction, refinement).
3) The approach that provides optimal exposure with minimal complication risk.
4) The endplate preparation technique that best represents the human procedure while creating sufficient space for the selected cage (size).
5) The need for additional spinal stabilization, which becomes more important with multiple levels.

Riccardo Audisio and colleagues mention *“Surgery, like sailing, is an art, and just as there is no “exact” way to set your sails, there is no exact way to tie your knots. However in surgery, as in sailing, experience and evidence are pivotal in improving our skills”.* [49] This reflects well that besides a step-by-step technique description, understanding of all above considerations is important when dealing with the IF animal model.

### 3.8 Relevant small ruminant surgical anatomy

**Figure 1:**
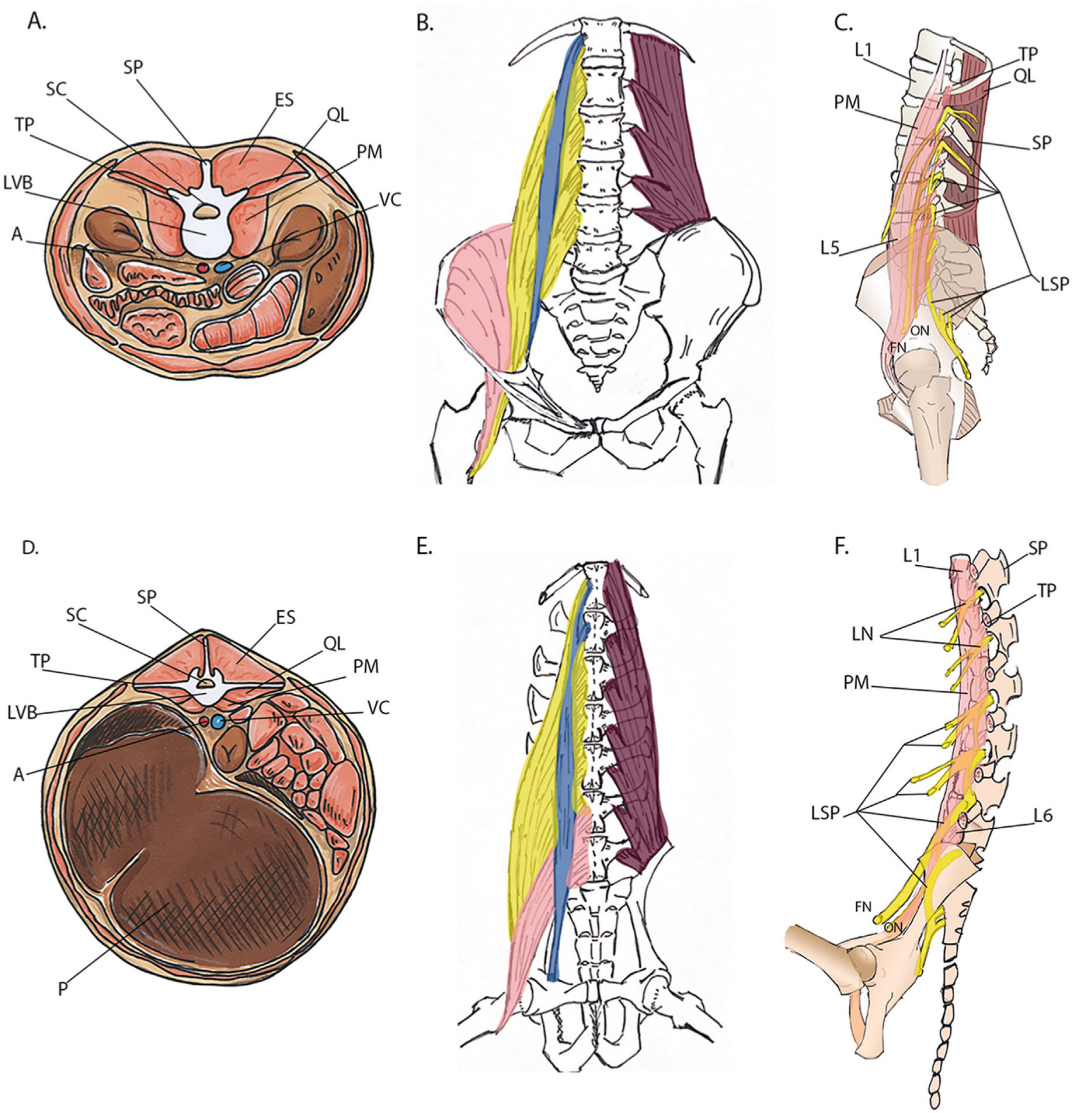
A Cross section of the human (1A) and goat (1D) abdomen at a lumbar level. In both cases the paraspinal musculature from posterior to anterior consists of the erector spinae muscles, QL and PM, but due to the different position of the TP in goats both the QL and PM cover the vertebral bodies. Fig. 1B and 1E show the anatomy of the QL (purple), PM (yellow), psoas minor (blue) and IM (pink) in coronal view. Figure 1C and 1F show the lumbar plexus anatomy. A= Aorta; VC; Vena Cava; P = Pouch; LVB = Lumbar vertebral body; TP=transverse process(es); SP = spinous process; SC=Spinal canal; ES= Erector spinae muscle; QL = Quadratus lumborum; PM = psoas muscle; IM = iliac muscle; FN = Femoral nerve; ON= Obturator nerve; LSP = lumbosacral plexus; LN = Lumbar nerves; L1=lumbar vertebra 1; L5 = lumbar vertebra 5; L6 = lumbar vertebra 6. (For interpretation of the references to color in this figure legend, the reader is referred to the Web version of this article.)

The small ruminant lumbar vertebral column differs from that in humans in many aspects (Fig. 1). In contrast to the central position in humans, the 6 lumbar ruminant vertebral bodies are much more dorsally located at small depth due to the lumbar kyphosis. The tall transverse processes (TP) are located anterior to the pedicles and originate from the vertebrae instead of the lamina (Fig. 1D). Anterior to the spine is a large trunk that fits the 4 stomachs that form one complex, referred to as the pouch [18,50].

Also, the spinal musculature is different. The quadratus lumborum (QL) proximally attaches to the thoracic vertebrae instead of the lowest ribs (Fig. 1E). At the lumbar levels it attaches more anteriorly to the base of the TP. Therefore, the QL is located directly lateral to the lumbar vertebral bodies just as the psoas muscles (PM) (Fig. 1D). Proximally, the IM in goats attaches to the lowest lumbar levels instead of the iliac crest (Fig. 1E). The psoas major and minor have the same function and anatomy as in humans. However, the relatively small vertebral bodies are almost fully covered (Fig. 1D).

As in humans the spine and spinal cord are supplied by segmental arteries that originate from the aorta and iliolumbar artery [51]. Although the position of the segmental vessels is relatively constant, the side branches show a high degree of variability that can complicate the surgical procedure.

In contrast to humans there is no cauda equina present at the lumbar levels, the spinal cord reaches as far as the lumbosacral junction [52]. Based on MRI and anatomic preparation of a goat specimen, the cord occupies about 80% of the spinal canal. In the human cervical spine the spinal cord occupies about 58% of the spinal canal and a spinal cord occupation rate of more than 75% is a criterion for developmental canal stenosis [53]. This means there is much less room for penetration of the canal as also found for dogs and pigs [54,55].

Plexus lesions on the other hand are more forgiving in small ruminants as motor innervation is only supplied from the fifth lumbar nerve and more caudally located nerves [56,57].(Fig. 1F) In humans the plexus is positioned within the psoas major muscle (1C, orange) and the femoral nerve (FN) and obturator nerve (ON) arise from the L2-L4 levels [58]. In goats the plexus is positioned medial to the psoas major muscle (1F, orange) and the FN and ON arise from the L5 and L6 nerve respectively.

### 3.9 Which and how many levels

Our search yielded fifty seven papers describing the IF model in small ruminants using a total of 1315 animals (Supplement A). In the papers that described animal gender (60%), mostly female animals were used (91%). The animals received either a one-level (44%), two-level (42%) or three-level procedure (5%)(Fig. 3). An intra-animal comparison allows for paired comparisons and therefore significantly reduces the number of animals. In our experience multilevel procedures are well tolerated by small ruminants. In 2–3 level procedures one untouched segment is left in between to limit reciprocal influence between operated segments.

**Figure 2:**
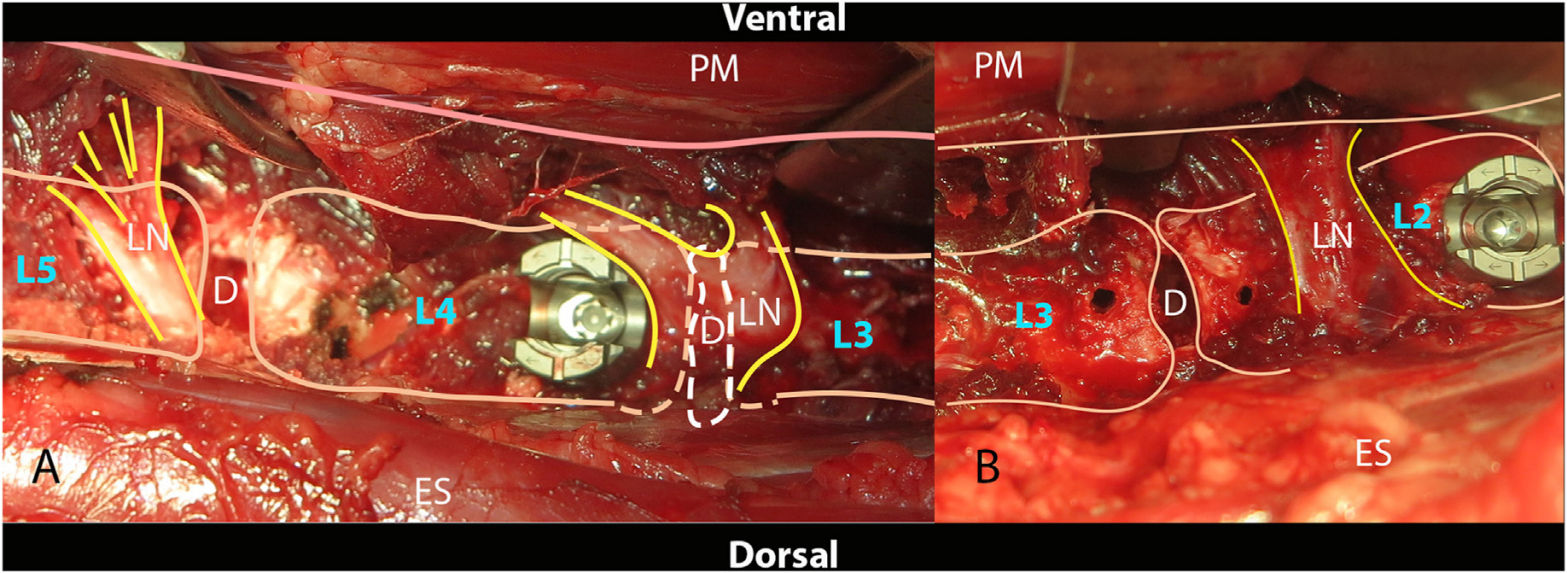
Peroperative photographs of A) L4-5 disc space B) L2-3 disc space(D) after IVD removal and screw preparation. At the L2-3 level the LN travels parallel to the disc space towards the ventral side of the psoas muscle (P). At the L3-4 and L4-5 levels the LN travel in a more caudal angle (partially) obstructing the disc spaces. IVD ​= ​intervertebral disc; LN ​= ​lumbar nerve; PM ​= ​psoas muscle; ES ​= ​Erector spinae muscle; L2 ​= ​Lumbar vertebra 2; L3 ​= ​Lumbar vertebra 3; L4 ​= ​lumbar vertebra 4; L5 ​= ​Lumbar vertebra 5.

**Figure 3:**
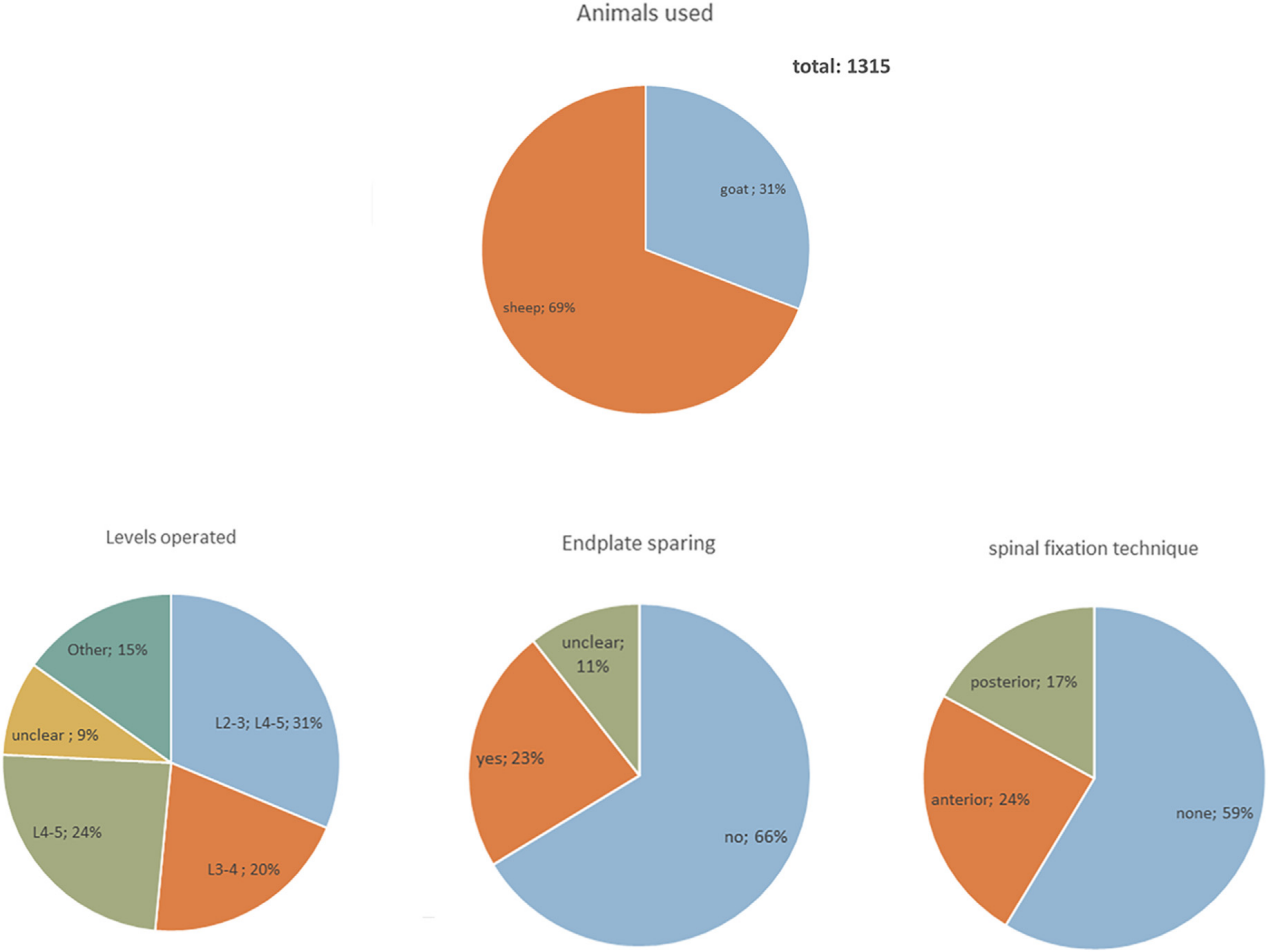
A summary of systematic literatur search on small ruminant IF: levels, endplate preparation and spinal fixation.

In most studies the L2-3 (36%), L3-4 (32%) or L4-5 (62%) levels are used(Fig. 3). In our experience the L1-2 and L2-3 levels are more convenient for an IF procedure than the lower lumbar levels which are covered by the lumbar plexus branches(Figure 1, Figure 2A). The more caudal procedure is furthermore restricted due to the presence of the psoas muscle and the iliac wings which may limit its exposure. If a caudal level is selected, ventral retraction should be kept to the absolute minimum to prevent overstretching of the nerves. After blunt preparation, the nerves can usually be pushed cranial to the IVD where they can be fixated behind a (steinmann) pin or pedicle probe. Detachment of the IM from the vertebrae is usually needed to place the pin/probe and for screw placement in case of spinal fixation.

### 3.10 Choosing the surgical approach

The lateral retroperitoneal approach is the most obvious for this procedure. In a posterior approach the spinal cord is encountered (the conus medullaris lies at S1-2) which considerably limits implant size and increases the risk of complications. Transperitoneal approaches are largely prohibited by the multiple stomachs that form one large impenetrable organ, occupying over 50% of the abdominal cavity [18,50].(Fig. 1D).

Mostly a left sided approach is used to avoid the liver and the frailty of the inferior vena cava. The tips of the TP can be palpated easily and reached superficially after the skin incision and outer muscle dissection. Deep dissection is just ventral to the TP until the level where the vertebral body and disc can be palpated [19,59]. From here the spinal column can be exposed both ventral (anterolateral approach) or dorsal to the psoas (retropsoas approach) [60]. In the anterolateral approach the spine is reached by retracting the psoas major and psoas minor muscles dorsally including the lumbar nerves, which is less invasive and allows for good visualization of the large vessels [19]. The trajectory of this approach is however suboptimal for IF procedures, as implantation is much more from anterior and directed towards the spinal canal. Also, the anterior longitudinal ligament (ALL) is easily damaged. In the retropsoas approach the psoas muscle is bluntly dissected just ventral to the base of the TP and retracted ventrally with a wide blade which provides good lateral access [59]. The position of the lumbar nerves ventral to the psoas major muscle and the smaller depth of the vertebrae facilitates retraction of the psoas major muscle ventrally without causing nerve damage, which would not be feasible in humans. A step-by-step description of this procedure is found in supplement C.

### 3.11 Intervertebral disc preparation

As the vertebral body of the goat has an hourglass shape the discs can be easily recognized as the broadest bulging parts of the spinal column. The disc spaces itself are biconvex with, in our study, a maximum height in the middle of 5,6-6,1 ​mm. Despite the local kyphosis, the anterior disc height (4-4.5 ​mm) exceeded that of the posterior disc height (3,5-4 ​mm), comparable to humans.

An important aspect is whether or not to maintain the endplates as this would better represent the human procedure. Endplate removal with a chisel or drill however, is more easy and can be standardized [61]. In the majority of animals used for IF models, endplate removal was performed(Fig. 3). In 2016 the first endplate sparing procedure for an implant efficacy study was described [62], that was subsequently used in several sheep studies. As incomplete removal of the CEP and the intervertebral disc tissue negatively influences fusion rates [37,63], specialized surgical equipment should be used. Despite thorough endplate preparations it's high likely some CEP will remain in any endplate sparing procedure. Therefore the authors feel the endplate sparing procedure has better translationability, compared to endplate removal, but it probably is a more challenging model to achieve IF. Specialized surgical equipment is also needed to allow implant positioning beyond the midline of the biconvex disc space, while preventing breach through the posterior longitudinal ligament(PLL).

### 3.12 Spinal stabilization

In clinical practice, spinal stabilization supplements the IF to increase the likelihood of fusion and reduce subsidence of the cage [64,65]. Also in small ruminants additional spinal stabilization has a positive effect on IF rates [66]. Cages aimed for stand-alone IF require specific traits that improve initial biomechanical stability [67]. Therefore stand-alone models should be reserved for cages designed with this specific purpose.

Only a minority (41%) of IF studies used spinal stabilization(Fig. 3). Most studies describe anterior stabilization techniques using one screw per vertebra adjacent to the operated level, connected with one rod (supplement A). Although improved stability occurs with these stabilizations [66], 2-point fixation is still suboptimal due to sagittal mobility. In our multilevel study we applied 2 screws at the end vertebrae (L2 and L5) and one in L3 and 4 one to optimize stability and minimize variability in the biomechanical environment.

## 4 Pitfalls and considerations regarding caprine anatomy

The surgical procedure should be performed by a trained surgeon to guarantee consistent quality. Although experienced spine surgeons performed the procedure in our study group, we experienced a steep learning curve. This was mainly for positioning of the animal and exposure of the lumbar spine. The average operative time decreased from 165 ​min to about 120 ​min during the timeframe of the study. Exposure may be complicated by the large bloated stomach of the goat. The pouch is encountered at every level and large stomach retractors and surgical assistance are needed. Pre-operative fasting for 12–24 ​h is suggested to reduce the bloating [68,69], but when looking at the animal's digestive physiology it's unlikely bloating can be prevented and fasting is potentially harmful for the animals [50].

In our study unfortunately 2 goats experienced a complete spinal cord injury (SCI). Autopsy revealed minimal (<1 ​mm) protrusion of an implant (once this was a screw and once this was a cage) in the canal. A major difference with humans is that the spinal cord reaches as far as the lumbosacral junction and occupies a relatively large percentage of the spinal canal. This imposes a much higher risk of SCI. In one case excessive blood loss occurred (1400 ​ml) after disruption of a segmental artery. Monopolar coagulation was insufficient and ligation of the vessel was needed. Other reports suggest bipolar coagulation. Except for the two SCI cases, the animals mobilized the day of the surgery. No wound infections were observed, suggesting that wound contamination by the goat itself or one of its peers can be prevented with standard wound closure and perioperative antibiotics.

## 5 Conclusion

The small ruminant lumbar IF model is a useful translational model to study the efficacy and function of interbody cages. Both the goat and sheep IF model show an adequate balance between the analogy to the human spine and its practical considerations for research purposes. IF models are often performed with female animals of different breeds, between-animal variability can be mitigated with internal controls, using multilevel procedures that are well tolerated. With the introduction of the endplate sparing model and appropriate spinal stabilization this model approximates the human situation as much as possible. However, even when performed by a trained surgeon, a learning curve has to be expected. A two-level, L1-2; L3-4, lateral retroperitoneal retropsoas approach gives adequate access to the spine for IF implantation and the lowest risk of complications. SCI is the most common complication in small ruminants IF models, as the spinal cord is extremely vulnerable and completely fills the spinal canal. Lumbar plexus damage on the other hand is uncommon especially when the L4-5 and L5-6 levels are omitted.

## Disclosure of funding sources

The research did not receive any specific grant from funding agencies in the public, commercial, or not-for-profit sectors.

## Declaration of competing interest

The authors declare that they have no known competing financial interests or personal relationships that could have appeared to influence the work reported in this paper.

## Appendix A Supplementary data

mmc1: Supplement A mmc1: Supplement A

mmc2: Supplement B mmc2: Supplement B

mmc3: Supplement C mmc3: Supplement C
