# Supplementary material for: Preclinical model for lumbar interbody fusion in small ruminants: Rationale and guideline
Source: J Orthop Translat. 2022 Nov 15;38:167–74. doi: 10.1016/j.jot.2022.10.006 (PMC9672886; doi:10.1016/j.jot.2022.10.006)
Supplement: mmc1: Supplement A [file mmc1.docx]

**Supplement A:**

**Systematic review ‘small ruminant IF model use’**

The electronic database Pubmed was searched for literature published through September 2020. Two sets of key search term were used in Pubmed: lumbar AND ("in vivo" OR "animal model") AND "fusion" AND (interbody OR cage OR anterior) and (lumbar AND ("in vivo" OR "animal model") AND (spine OR intervertebral disc) AND (pig OR porcine OR dog OR canine OR sheep OR ovine OR goat OR caprine OR cow OR cattle OR bovine OR primate OR primates) AND fusion). After removal of duplicates, the identified references were assessed for eligibility based on title and abstract by 1 reviewer (AD) using Rayyan QCRI. Only in vivo studies that used a lumbar IF procedure in small ruminants were included. The full-text of potential papers was retrieved and checked for inclusions.


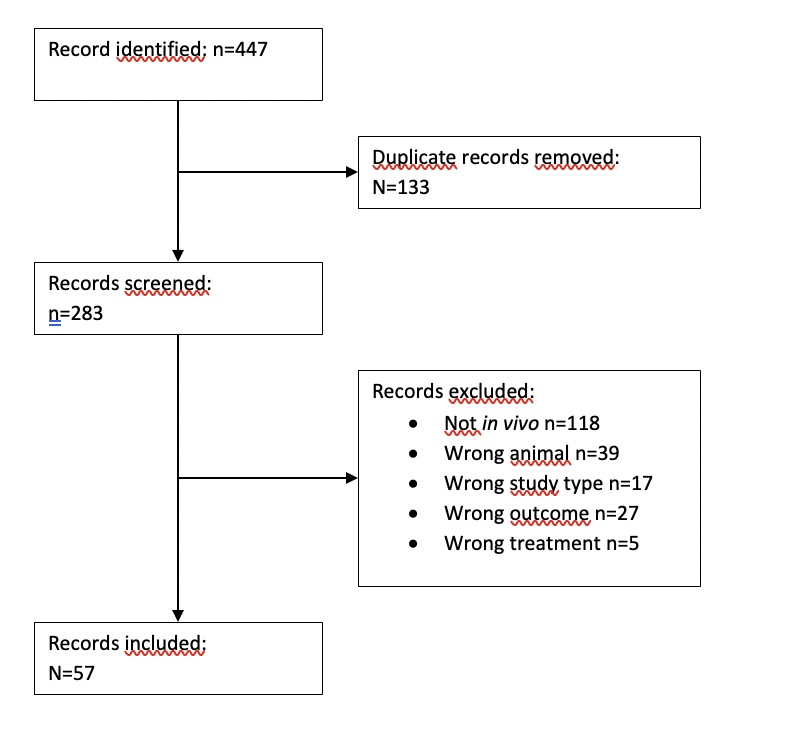


The following data were extracted from the included papers: year of publication, first author, species used, approach used, levels operated, endplate preparation and fixation technique.

| Table 1: Summary of studies investigating interbody fusion in a goat IF model (in order of publication year) | | | | |
| --- | --- | --- | --- | --- |
| **Author (year)** | **Number of Animals** | **Level(s) operated** | **Endplate Sparing (Yes/No)** | **Spinal fixation** |
| Brantigan et al. (1994)[1] | 27 | L4-5 | No | None |
| Pintar et al. (1994)[2] | 14 | Unclear | No | None |
| Mooney et al (1998)[3] | 9 | L4; L5; L6 | N/A | None |
| Van Dijk et al. (2002)[4] | 18 | L3-4 | No | None |
| Van Dijk et al. (2002)[5] | 36 | L3-4 | No | None |
| Wuisman et al. (2002)[6] | 36 | L3-4 | No | None |
| Krijnen et al. (2006)[7] | 35 | L3-4 | No | A subgroup received anterior screw fixation connecting 2 vertebrae using 2 screws and a short rod |
| Smit et al. (2006)[8] | 78 | L3-4 | No | A subgroup received anterior screw fixation connecting 2 vertebrae using 2 screws and a short rod |
| Mullender et al. (2007)[9] | 20 | L3-4 | No | None |
| Smit et al. (2007)[10] | 7 | L3-4 | No | None |
| Kroeze et al. (2013)[11] | 20 | L1-2; L3-4 | No | None |
| Kroeze et al. (2015)[12] | 36 | L1-2; L3-4 | No | None |
| Xiao et al. (2015)[13] | 41 | L4-5 | No | None |
| MacEwan et al. (2016)[14] | 6 | L4-5 | Yes | Posterior pedicle screw fixation |
| Huang et al. (2018)[15] | 15 | L4-5 | No | None |
| Kersten et al. (2019)[16] | 8 | L1-2; L3-4 | No | Anterior screw fixation connecting 2 vertebrae using 2 screws and a short rod |

| Table 2: Summary of studies investigating interbody fusion in a sheep IF model (in order of publication year) | | | | |
| --- | --- | --- | --- | --- |
| **Author (year)** | **Number of Animals** | **Level(s) operated** | **Endplate Sparing (Yes/No)** | **Spinal fixation** |
| Sandhu et al. (1996)[17] | 20 | L4-5 | No | None |
| Steffen et al. (2000)[18] | 54 | L2-3; L4-5 | Yes | Posterior pedicle screw fixation combined with posterolateral fusion |
| Toth et al. (2000)[19] | 22 | L4-5 | No | None |
| Magin et al. (2001)[20] | 33 | L4-5 | Yes | Posterior pedicle screw fixation |
| Steffen et al. (2001)[21] | 54 | L2-3; L4-5 | Yes | None |
| Blattert et al. (2002)[22] | 14 | L4-5 | Yes | Posterior pedicle screw fixation |
| Sandhu et al. (2002)[23] | 12 | L4-5 | No | None |
| Toth et al. (2002)[24] | 25 | L4-5 | No | None |
| Assad et al. (2003)[25] | 16 | L2-3; L4-5 | No | None |
| Takahata et al. (2003)[26] | 20 | L2-3; L4-5 | No | Anterior screw fixation connecting 2 vertebrae using 2 screws and a short rod |
| Likibi et al. (2005)[27] | 18 | L2-3; L4-5 | No | None |
| Takahata et al. (2005)[28] | 24 | L2-3; L4-5 | No | Anterior screw fixation connecting 2 vertebrae using 2 screws and a short rod |
| Lazennec et al. (2006)[29] | 16 | L3-4 | No | None |
| Toth et al. (2006)[30] | 13 | L4-5 | No | None |
| Ito et al. (2007)[31] | 21 | L2-3; L4-5 | No | Anterior screw fixation connecting 2 vertebrae using 2 screws and a short rod |
| Manunta et al. (2008)[32] | 5 | L3-4 | No | None |
| Strohm et al. (2008)[33] | 7 | L3-4 | Unclear | Anterior angular stable locking plate fixation |
| Cunningham et al. (2009)[34] | 20 | L2-3; L4-5 | No | Posterior fixation not specified |
| Qian et al. (2009)[35] | 28 | L3-4; L4-5 | No | Anterior Bone staples |
| Sherman et al. (2010)[36] | 6 | L3-4; L4-5 | Unclear | None |
| Gu et al. (2011)[37] | 24 | L1-2; L3-4; L5-6 | No | None |
| Siu et al. (2011)[38] | 18 | L3-4; L5-6 | No | None |
| Oehme et al. (2012)[39] | 95 | unclear | Unclear | None |
| Solchaga et al. (2012)[40] | 22 | L2-3; L4-5 | No | None |
| Hong et al. (2014)[41] | 15 | Unclear | Unclear | None |
| Chen et al. (2015)[42] | 30 | L1-2; L3-4; L5-6 | No | None |
| Yamada et al. (2015)[43] | 12 | L2-3; L4-5 | Yes | Anterior screw fixation connecting 2 vertebrae using 2 screws and a short rod |
| Bae et al. (2016)[44] | 21 | L2-3; L4-5 | Yes | None |
| Pelletier et al. (2016)[45] | 9 | L2-3; L3-4 | Yes | None |
| Wheeler et al. (2016)[46] | 32 | L4-5 | No | Posterior pedicle screw fixation |
| McGilvray et al. (2017)[47] | 32 | L2-3; L4-5 | No | Anterior fixation not specified |
| Pan et al. (2017)[48] | 6 | L3-4; L5-6 | No | None |
| Qian et al. (2017)[49] | 8 | L3-4; L4-5 | Yes | Anterior bone staples |
| McGilvray et al. (2018)[50] | 27 | L2-3; L4-5 | No | Anterior fixation not specified |
| Aihara et al. (2019)[51] | 8 | L2-3; L4-5 | Unclear | Anterior fixation not specified |
| Gunzburg et al. (2019)[52] | 14 | L4-5 | Yes | None |
| Walsh et al. (2019)[53] | 8 | L4-5 | Yes | Posterior pedicle screw fixation |
| Cohen et al. (2020)[54] | 12 | L2-3; L4-5 | No | A subgroup received posterior fixation not specified |
| Walsh et al. (2020)[55] | 40 | L4-5 | Yes | Posterior pedicle screw fixation |
| Loenen et al. (2021)[56] | 30 | L2-3; L4-5 | Yes | Anterior plastic intervertebral plate with 4 metal screws |
| Van Horn et al. (2021)[57] | 18 | L2-3; L4-5 | No | Posterior pedicle screw fixation |

[1] J. W. Brantigan, P. C. McAfee, B. W. Cunningham, H. Wang, en C. M. Orbegoso, ‘Interbody lumbar fusion using a carbon fiber cage implant versus allograft bone. An investigational study in the Spanish goat’, *Spine*, vol. 19, nr. 13, pp. 1436–1444, jul. 1994, doi: 10.1097/00007632-199407000-00002.

[2] F. A. Pintar *e.a.*, ‘Fusion rate and biomechanical stiffness of hydroxylapatite versus autogenous bone grafts for anterior discectomy. An in vivo animal study’, *Spine*, vol. 19, nr. 22, pp. 2524–2528, nov. 1994, doi: 10.1097/00007632-199411001-00006.

[3] V. Mooney, J. B. Massie, B. I. Lind, J. H. Rah, S. Negri, en R. E. Holmes, ‘Comparison of hydroxyapatite granules to autogenous bone graft in fusion cages in a goat model’, *Surg Neurol*, vol. 49, nr. 6, pp. 628–633; discussion 633-634, jun. 1998, doi: 10.1016/s0090-3019(98)00049-4.

[4] M. van Dijk, D. C. Tunc, T. H. Smit, P. Higham, E. H. Burger, en P. I. J. M. Wuisman, ‘In vitro and in vivo degradation of bioabsorbable PLLA spinal fusion cages’, *Journal of Biomedical Materials Research*, vol. 63, nr. 6, pp. 752–759, 2002, doi: https://doi.org/10.1002/jbm.10466.

[5] M. van Dijk, T. H. Smit, E. H. Burger, en P. I. Wuisman, ‘Bioabsorbable poly-L-lactic acid cages for lumbar interbody fusion: three-year follow-up radiographic, histologic, and histomorphometric analysis in goats’, *Spine (Phila Pa 1976)*, vol. 27, nr. 23, pp. 2706–2714, dec. 2002, doi: 10.1097/00007632-200212010-00010.

[6] P. I. J. M. Wuisman, M. van Dijk, en T. H. Smit, ‘Resorbable cages for spinal fusion: an experimental goat model’, *J Neurosurg*, vol. 97, nr. 4 Suppl, pp. 433–439, nov. 2002, doi: 10.3171/spi.2002.97.4.0433.

[7] M. R. Krijnen, M. G. Mullender, T. H. Smit, V. Everts, en P. I. J. M. Wuisman, ‘Radiographic, histologic, and chemical evaluation of bioresorbable 70/30 poly-L-lactide-CO-D, L-lactide interbody fusion cages in a goat model’, *Spine*, vol. 31, nr. 14, pp. 1559–1567, jun. 2006, doi: 10.1097/01.brs.0000221984.12004.3b.

[8] T. H. Smit, M. R. Krijnen, M. van Dijk, en P. I. J. M. Wuisman, ‘Application of polylactides in spinal cages: studies in a goat model’, *J Mater Sci Mater Med*, vol. 17, nr. 12, pp. 1237–1244, dec. 2006, doi: 10.1007/s10856-006-0597-5.

[9] M. G. Mullender, M. R. Krijnen, M. N. Helder, T. H. Smit, V. Everts, en P. I. J. M. Wuisman, ‘Lumbar body fusion with a bioresorbable cage in a goat model is delayed by the use of a carboxymethylcellulose-stabilized collagenous rhOP-1 device’, *J. Orthop. Res.*, vol. 25, nr. 1, pp. 132–141, jan. 2007, doi: 10.1002/jor.20285.

[10] T. H. Smit, K. A. Thomas, R. J. W. Hoogendoorn, G. J. Strijkers, M. N. Helder, en P. I. J. M. Wuisman, ‘Sterilization and strength of 70/30 polylactide cages: e-beam versus ethylene oxide’, *Spine (Phila Pa 1976)*, vol. 32, nr. 7, pp. 742–747, apr. 2007, doi: 10.1097/01.brs.0000259057.94986.3b.

[11] R. J. Kroeze, A. J. van der Veen, B. J. van Royen, R. A. Bank, M. N. Helder, en T. H. Smit, ‘Relation between radiological assessment and biomechanical stability of lumbar interbody fusion in a large animal model’, *Eur Spine J*, vol. 22, nr. 12, pp. 2731–2739, dec. 2013, doi: 10.1007/s00586-013-3003-2.

[12] R. J. Kroeze *e.a.*, ‘Spinal fusion using adipose stem cells seeded on a radiolucent cage filler: a feasibility study of a single surgical procedure in goats’, *Eur Spine J*, vol. 24, nr. 5, pp. 1031–1042, mei 2015, doi: 10.1007/s00586-014-3696-x.

[13] J. Xiao, Y.-C. Huang, S. K. L. Lam, en K. D. K. Luk, ‘Surgical technique for lumbar intervertebral disc transplantation in a goat model’, *Eur Spine J*, vol. 24, nr. 9, pp. 1951–1958, sep. 2015, doi: 10.1007/s00586-014-3631-1.

[14] M. R. MacEwan, M. R. Talcott, D. W. Moran, en E. C. Leuthardt, ‘Novel spinal instrumentation to enhance osteogenesis and fusion: a preliminary study’, *J Neurosurg Spine*, vol. 25, nr. 3, pp. 318–327, sep. 2016, doi: 10.3171/2016.1.SPINE13979.

[15] Y.-C. Huang, J. Xiao, V. Y. Leung, W. W. Lu, Y. Hu, en K. D. K. Luk, ‘Lumbar intervertebral disc allograft transplantation: the revascularisation pattern’, *Eur Spine J*, vol. 27, nr. 3, pp. 728–736, mrt. 2018, doi: 10.1007/s00586-017-5419-6.

[16] R. F. M. R. Kersten *e.a.*, ‘Comparison of polyetheretherketone versus silicon nitride intervertebral spinal spacers in a caprine model’, *J Biomed Mater Res B Appl Biomater*, vol. 107, nr. 3, pp. 688–699, apr. 2019, doi: 10.1002/jbm.b.34162.

[17] H. S. Sandhu *e.a.*, ‘Distractive properties of a threaded interbody fusion device. An in vivo model’, *Spine (Phila Pa 1976)*, vol. 21, nr. 10, pp. 1201–1210, mei 1996, doi: 10.1097/00007632-199605150-00013.

[18] T. Steffen, D. Marchesi, en M. Aebi, ‘Posterolateral and anterior interbody spinal fusion models in the sheep’, *Clin. Orthop. Relat. Res.*, nr. 371, pp. 28–37, feb. 2000, doi: 10.1097/00003086-200002000-00004.

[19] J. M. Toth, H. B. Seim, J. D. Schwardt, W. B. Humphrey, J. A. Wallskog, en A. S. Turner, ‘Direct current electrical stimulation increases the fusion rate of spinal fusion cages’, *Spine*, vol. 25, nr. 20, pp. 2580–2587, okt. 2000, doi: 10.1097/00007632-200010150-00007.

[20] M. N. Magin en G. Delling, ‘Improved lumbar vertebral interbody fusion using rhOP-1: a comparison of autogenous bone graft, bovine hydroxylapatite (Bio-Oss), and BMP-7 (rhOP-1) in sheep’, *Spine*, vol. 26, nr. 5, pp. 469–478, mrt. 2001, doi: 10.1097/00007632-200103010-00009.

[21] T. Steffen, T. Stoll, T. Arvinte, en R. K. Schenk, ‘Porous tricalcium phosphate and transforming growth factor used for anterior spine surgery’, *Eur Spine J*, vol. 10 Suppl 2, pp. S132-140, okt. 2001, doi: 10.1007/s005860100325.

[22] T. R. Blattert, G. Delling, en A. Weckbach, ‘[Pediculoscopic assisted transpedicular spongioplasty for interbody fusion of the lumbar spine. An animal experiment study of the sheep model]’, *Unfallchirurg*, vol. 105, nr. 8, pp. 680–687, aug. 2002.

[23] H. S. Sandhu *e.a.*, ‘Histologic evaluation of the efficacy of rhBMP-2 compared with autograft bone in sheep spinal anterior interbody fusion’, *Spine*, vol. 27, nr. 6, pp. 567–575, mrt. 2002, doi: 10.1097/00007632-200203150-00003.

[24] J. M. Toth *e.a.*, ‘Evaluation of 70/30 poly (L-lactide-co-D,L-lactide) for use as a resorbable interbody fusion cage’, *J. Neurosurg.*, vol. 97, nr. 4 Suppl, pp. 423–432, nov. 2002, doi: 10.3171/spi.2002.97.4.0423.

[25] M. Assad *e.a.*, ‘Porous titanium-nickel for intervertebral fusion in a sheep model: part 1. Histomorphometric and radiological analysis’, *J. Biomed. Mater. Res. Part B Appl. Biomater.*, vol. 64, nr. 2, pp. 107–120, feb. 2003, doi: 10.1002/jbm.b.10530.

[26] M. Takahata *e.a.*, ‘Bone ingrowth fixation of artificial intervertebral disc consisting of bioceramic-coated three-dimensional fabric’, *Spine (Phila Pa 1976)*, vol. 28, nr. 7, pp. 637–644; discussion 644, apr. 2003, doi: 10.1097/01.BRS.0000051918.47287.3E.

[27] F. Likibi, M. Assad, C. Coillard, G. Chabot, en C.-H. Rivard, ‘[Bone integration and apposition of porous and non porous metallic orthopaedic biomaterials]’, *Ann Chir*, vol. 130, nr. 4, pp. 235–241, apr. 2005, doi: 10.1016/j.anchir.2004.12.006.

[28] M. Takahata *e.a.*, ‘An investigational study on the healing process of anterior spinal arthrodesis using a bioactive ceramic spacer and the change in load-sharing of spinal instrumentation’, *Spine (Phila Pa 1976)*, vol. 30, nr. 8, pp. E195-203, apr. 2005, doi: 10.1097/01.brs.0000158958.54575.0c.

[29] J. Y. Lazennec, A. Madi, M. A. Rousseau, B. Roger, en G. Saillant, ‘Evaluation of the 96/4 PLDLLA polymer resorbable lumbar interbody cage in a long term animal model’, *Eur Spine J*, vol. 15, nr. 10, pp. 1545–1553, okt. 2006, doi: 10.1007/s00586-006-0145-5.

[30] J. M. Toth, M. Wang, B. T. Estes, J. L. Scifert, H. B. Seim, en A. S. Turner, ‘Polyetheretherketone as a biomaterial for spinal applications’, *Biomaterials*, vol. 27, nr. 3, pp. 324–334, jan. 2006, doi: 10.1016/j.biomaterials.2005.07.011.

[31] M. Ito, Y. Kotani, Y. Hojo, K. Abumi, T. Kadosawa, en A. Minami, ‘Evaluation of hydroxyapatite ceramic vertebral spacers with different porosities and their binding capability to the vertebral body: an experimental study in sheep’, *J Neurosurg Spine*, vol. 6, nr. 5, pp. 431–437, mei 2007, doi: 10.3171/spi.2007.6.5.431.

[32] M. L. Manunta *e.a.*, ‘Lumbar interbody expanding cage. A preliminary study on an animal model’, *Vet Comp Orthop Traumatol*, vol. 21, nr. 4, pp. 382–384, 2008.

[33] P. C. Strohm, D. Kubosch, T. A. Bley, C. M. Sprecher, N. P. Südkamp, en S. Milz, ‘Detection of bone graft failure in lumbar spondylodesis: spatial resolution with high-resolution peripheral quantitative CT’, *AJR Am J Roentgenol*, vol. 190, nr. 5, pp. 1255–1259, mei 2008, doi: 10.2214/AJR.07.2701.

[34] B. W. Cunningham *e.a.*, ‘Ceramic granules enhanced with B2A peptide for lumbar interbody spine fusion: an experimental study using an instrumented model in sheep’, *J Neurosurg Spine*, vol. 10, nr. 4, pp. 300–307, apr. 2009, doi: 10.3171/2009.1.SPINE08565.

[35] Y. Qian *e.a.*, ‘Natural bone collagen scaffold combined with autologous enriched bone marrow cells for induction of osteogenesis in an ovine spinal fusion model’, *Tissue Eng Part A*, vol. 15, nr. 11, pp. 3547–3558, nov. 2009, doi: 10.1089/ten.TEA.2009.0076.

[36] B. P. Sherman *e.a.*, ‘Evaluation of ABM/P-15 versus autogenous bone in an ovine lumbar interbody fusion model’, *Eur Spine J*, vol. 19, nr. 12, pp. 2156–2163, dec. 2010, doi: 10.1007/s00586-010-1546-z.

[37] Y. Gu, L. Chen, H.-L. Yang, Z.-P. Luo, en T.-S. Tang, ‘Evaluation of an injectable silk fibroin enhanced calcium phosphate cement loaded with human recombinant bone morphogenetic protein-2 in ovine lumbar interbody fusion’, *J Biomed Mater Res A*, vol. 97, nr. 2, pp. 177–185, mei 2011, doi: 10.1002/jbm.a.33018.

[38] R. K. Siu *e.a.*, ‘Nell-1 protein promotes bone formation in a sheep spinal fusion model’, *Tissue Eng Part A*, vol. 17, nr. 7–8, pp. 1123–1135, apr. 2011, doi: 10.1089/ten.TEA.2010.0486.

[39] D. Oehme *e.a.*, ‘Lateral surgical approach to lumbar intervertebral discs in an ovine model’, *ScientificWorldJournal*, vol. 2012, p. 873726, 2012, doi: 10.1100/2012/873726.

[40] L. A. Solchaga *e.a.*, ‘Augment bone graft products compare favorably with autologous bone graft in an ovine model of lumbar interbody spine fusion’, *Spine*, vol. 37, nr. 8, pp. E461-467, apr. 2012, doi: 10.1097/BRS.0b013e31823b01dc.

[41] X. Hong, X. Wu, S. Zhuang, J. Bao, en R. Shi, ‘New cage for posterior minimally invasive lumbar interbody fusion: a study in vitro and in vivo’, *Orthop Surg*, vol. 6, nr. 1, pp. 47–53, feb. 2014, doi: 10.1111/os.12083.

[42] Chen L, Liu HL, Gu Y, Feng Y, en Yang HL, ‘Lumbar interbody fusion with porous biphasic calcium phosphate enhanced by recombinant bone morphogenetic protein-2/silk fibroin sustained-released microsphere: an experimental study on sheep model.’, *J Mater Sci Mater Med*, vol. 26, nr. 3, p. 126, 2015.

[43] K. Yamada, M. Ito, T. Akazawa, M. Murata, T. Yamamoto, en N. Iwasaki, ‘A preclinical large animal study on a novel intervertebral fusion cage covered with high porosity titanium sheets with a triple pore structure used for spinal fusion’, *Eur Spine J*, vol. 24, nr. 11, pp. 2530–2537, nov. 2015, doi: 10.1007/s00586-015-4047-2.

[44] H. W. Bae *e.a.*, ‘Transient Local Bone Remodeling Effects of rhBMP-2 in an Ovine Interbody Spine Fusion Model’, *J Bone Joint Surg Am*, vol. 98, nr. 24, pp. 2061–2070, dec. 2016, doi: 10.2106/JBJS.16.00345.

[45] M. H. Pelletier, N. Cordaro, V. M. Punjabi, M. Waites, A. Lau, en W. R. Walsh, ‘PEEK Versus Ti Interbody Fusion Devices: Resultant Fusion, Bone Apposition, Initial and 26-Week Biomechanics’, *Clin Spine Surg*, vol. 29, nr. 4, pp. E208-214, mei 2016, doi: 10.1097/BSD.0b013e31826851a4.

[46] D. L. Wheeler, D. C. Fredericks, R. F. Dryer, en H. W. Bae, ‘Allogeneic mesenchymal precursor cells (MPCs) combined with an osteoconductive scaffold to promote lumbar interbody spine fusion in an ovine model’, *Spine J*, vol. 16, nr. 3, pp. 389–399, mrt. 2016, doi: 10.1016/j.spinee.2015.08.019.

[47] K. C. McGilvray *e.a.*, ‘Evaluation of a polyetheretherketone (PEEK) titanium composite interbody spacer in an ovine lumbar interbody fusion model: biomechanical, microcomputed tomographic, and histologic analyses’, *Spine J*, vol. 17, nr. 12, pp. 1907–1916, 2017, doi: 10.1016/j.spinee.2017.06.034.

[48] H. C. Pan *e.a.*, ‘Cyst-Like Osteolytic Formations in Recombinant Human Bone Morphogenetic Protein-2 (rhBMP-2) Augmented Sheep Spinal Fusion’, *Am. J. Pathol.*, vol. 187, nr. 7, pp. 1485–1495, jul. 2017, doi: 10.1016/j.ajpath.2017.03.010.

[49] Y. Qian, Z. Lin, C. Jin, X. Zhao, en M. Zheng, ‘A Less-Invasive Retroperitoneal Lumbar Approach: Animal Feasibility Study and Primary Clinical Study’, *Clin Spine Surg*, vol. 30, nr. 6, pp. 251–258, jul. 2017, doi: 10.1097/BSD.0000000000000252.

[50] K. C. McGilvray *e.a.*, ‘Bony ingrowth potential of 3D-printed porous titanium alloy: a direct comparison of interbody cage materials in an in vivo ovine lumbar fusion model’, *Spine J*, vol. 18, nr. 7, pp. 1250–1260, 2018, doi: 10.1016/j.spinee.2018.02.018.

[51] H. Aihara, J. Zider, G. Fanton, en T. Duerig, ‘Combustion Synthesis Porous Nitinol for Biomedical Applications’, *International Journal of Biomaterials*, vol. 2019, pp. 1–11, apr. 2019, doi: 10.1155/2019/4307461.

[52] R. Gunzburg *e.a.*, ‘Does nanoscale porous titanium coating increase lumbar spinal stiffness of an interbody fusion cage? An in vivo biomechanical analysis in an ovine model’, *Clin Biomech (Bristol, Avon)*, vol. 67, pp. 187–196, jul. 2019, doi: 10.1016/j.clinbiomech.2019.04.024.

[53] W. R. Walsh, M. H. Pelletier, T. Wang, V. Lovric, P. Morberg, en R. J. Mobbs, ‘Does implantation site influence bone ingrowth into 3D-printed porous implants?’, *Spine J*, jun. 2019, doi: 10.1016/j.spinee.2019.06.020.

[54] D. J. Cohen, L. Ferrara, M. B. Stone, Z. Schwartz, en B. D. Boyan, ‘Cell and Tissue Response to Polyethylene Terephthalate Mesh Containing Bone Allograft in Vitro and in Vivo’, *Int J Spine Surg*, vol. 14, nr. s3, pp. S121–S132, dec. 2020, doi: 10.14444/7135.

[55] W. R. Walsh, M. Pelletier, D. Wills, T. Wang, S. Bannigan, en F. Vizesi, ‘Undercut macrostructure topography on and within an interbody cage improves biomechanical stability and interbody fusion’, *Spine J*, vol. 20, nr. 11, pp. 1876–1886, nov. 2020, doi: 10.1016/j.spinee.2020.06.023.

[56] Loenen ACY *e.a.*, ‘Peptide Enhanced Bone Graft Substitute Presents Improved Short-Term Increase in Bone Volume and Construct Stiffness Compared to Iliac Crest Autologous Bone in an Ovine Lumbar Interbody Fusion Model.’, *Global Spine J*, p. 2192568220979839, 2021.

[57] M. R. Van Horn *e.a.*, ‘Comparison of 3D-printed titanium-alloy, standard titanium-alloy, and PEEK interbody spacers in an ovine model’, *Spine J*, pp. S1529-9430(21)00271–0, mei 2021, doi: 10.1016/j.spinee.2021.05.018.
