# Supplementary material for: Preclinical model for lumbar interbody fusion in small ruminants: Rationale and guideline
Source: J Orthop Translat. 2022 Nov 15;38:167–74. doi: 10.1016/j.jot.2022.10.006 (PMC9672886; doi:10.1016/j.jot.2022.10.006)
Supplement: mmc2: Supplement B [file mmc2.docx]

**Supplement B:**

**Housing and Husbandry:**

The animals were housed according to the Dutch and European animal welfare required parameters and AAALAC international accreditation.[1], [2] The animals were group housed unless study procedures, medical treatment or animal temperament necessitated individual housing. The kennels are designed in a fashion that even when animals are housed individually they can see, hear and smell each other at all times. All animals were kept in individual housing the first days after surgery until sufficient recovery to prevent hierarchic fights. One animal was placed in individual housing later in the study after harassment by its peers. This animal remained in individual housing for the remainder of the study, because the peers didn’t allow her presence back in the group.

**Animal Care and welfare monitoring:**

The goats were fed 200-300 grams of goat grain and muesli per day provided in their pens. Hay and water was provided *ab libitum*. All animals acclimatized for two weeks. One day before surgery the animals were weighed, and a Buprenorphine patch (35 microgram/hour) was applied under the tail. The Buprenorphine patch was continued 4-7 days after surgery. The first three days postoperatively, Meloxicam (0.4 mg/kg) was administered subcutaneously on a daily basis. Every day the general health of each animal was checked according to general veterinary standards . Deviations in behavior, posture, walk, feeding condition, food intake, water intake, state, clinical abnormalities and wound healing were reported to the primary researcher and to the on call veterinarian to treat the animal if needed

In three animals post-surgical abnormalities were detected. Two animals had a postoperative paraplegia for which Dexamethasone was administered without effect and therefore early euthanasia followed. Necropsy was performed in both animals to determine the cause of the neurologic deficit. In both cases iatrogenic damage to the spinal cord resulted in severe spinal cord injury (SCI).

One animal showed behavioral changes and reduced food intake without significant weight loss. Bowel complaints due to post-operative morphine use was suspected and therefore the animal received Buscopan Compositum with good clinical result.

**Anesthetic procedure:**

The peri-operative procedures have been performed according to the code of practice.[3] The animal was sedated with an intravenous injection of Detomidine (0.04 mg/kg). Initially the general anesthesia was induced with an intravenous injection of Propofol (2 mg/kg). An endotracheal tube was placed and ophthalmic ointment applied. Initially maintenance of the general anesthesia was achieved with intravenous Propofol (10 mg/kg/h), Sufentanil (0.007mg/kg/h) and Cisatracurium (0.09 mg/kg/h). During the study the Sufentanil was replaced for Remifentanil (0.03 mg/kg/h), because this significantly reduced the time the animal remained sedated post-operatively. Furthermore the propofol was replaced by midazolam (0.8 mg/kg/h) and isoflurane inhalation was added to prevent spontaneous breathing. An intravenous injection of meloxicam (0.5 mg/kg) and Amocycillin/Clavulanic acid (10 mg/kg) was administered as analgesia and antibiotic prophylaxis respectively.

**Euthanasia:**

General deep sedation was induced with an intravenous injection of Detomidine. When sufficient level of anesthesia was reached an intravenous lethal dose of pentobarbital was administered. The dosages of drugs were based on animal weight and the veterinarian’s preference to the animal’s individual needs.

[1] Directive 2010/63/EU of the European Parliament and of the Council of 22 September 2010 on the protection of animals used for scientific purposes Text with EEA relevance, vol. 276. 2010. Geraadpleegd: 10 juni 2022. [Online]. Beschikbaar op: http://data.europa.eu/eli/dir/2010/63/oj/eng

[2] ‘wetten.nl - Regeling - Wet op de dierproeven - BWBR0003081’. https://wetten.overheid.nl/BWBR0003081/2021-07-01 (geraadpleegd 28 juni 2022).

[3] N. en V. Ministerie van Landbouw, ‘Code of Practive Voorkómen, herkennen en bestrijden van pijn bij proefdieren - Rapport - Nationaal Comité advies dierproevenbeleid’, 26 mei 2016. https://www.ncadierproevenbeleid.nl/documenten/rapport/2016/5/26/cop-pijn-proefdieren (geraadpleegd 28 juni 2022).
