# Supplementary material for: Preclinical model for lumbar interbody fusion in small ruminants: Rationale and guideline
Source: J Orthop Translat. 2022 Nov 15;38:167–74. doi: 10.1016/j.jot.2022.10.006 (PMC9672886; doi:10.1016/j.jot.2022.10.006)
Supplement: mmc3: Supplement C [file mmc3.docx]

**Supplement C:**

**Step-by-step surgical approach:**

- Place the animal in a right lateral decubitus position that allows 90 degrees lateral fluoroscopic imaging.
- Position the mouth below the throatlatch to prevent complications from salivation respiration(fig 1A). Use taping and bean bags to secure the position.


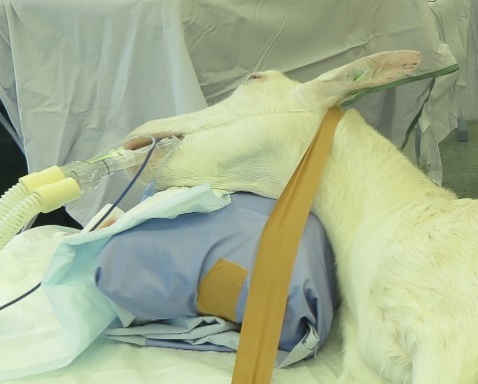

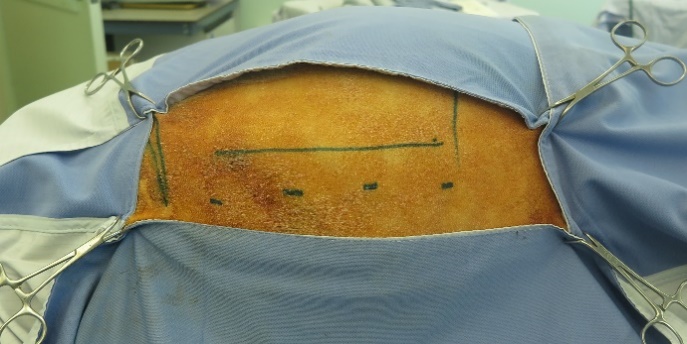


B

A

Figure 1: A) Photograph mouth above throatlatch to prevent saliva aspiration B) Photograph preparation surgical field

- Prepare and drape the lumbar spine in a sterile fashion, exposing the spinous processes and the left flank. (fig 1B)
- Make an incision alongside the transverse processes (TP) from the L1 distal endplate extending to the L6 proximal endplate.
- Use subcutaneous dissection to expose the lateral wall musculature (fig 2) and make a straight incision in line with the skin incision through the lateral wall musculature; avoid entering the peritoneum.


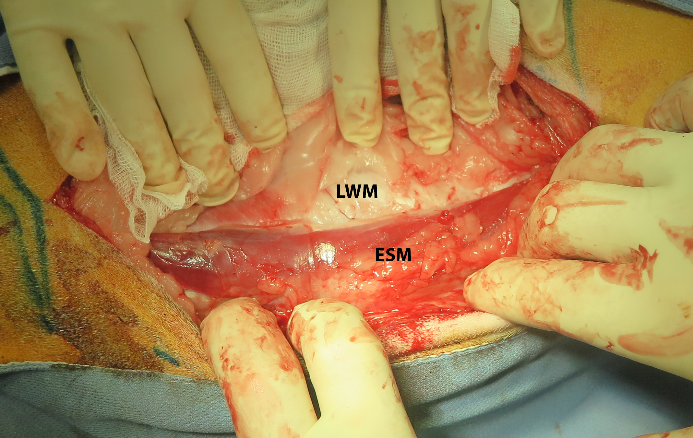


Figure 2: Photograph tension on the lateral wall musculature (LWM). Both the LWM and erector spinae muscle (ESM) attach to the transverse processes.

- Retract the retroperitoneal fat ventrally using large abdominal retractors held by a surgical assistant and palpate the psoas muscle. (fig 3) Aim for the lateral tip of the TP as a landmark


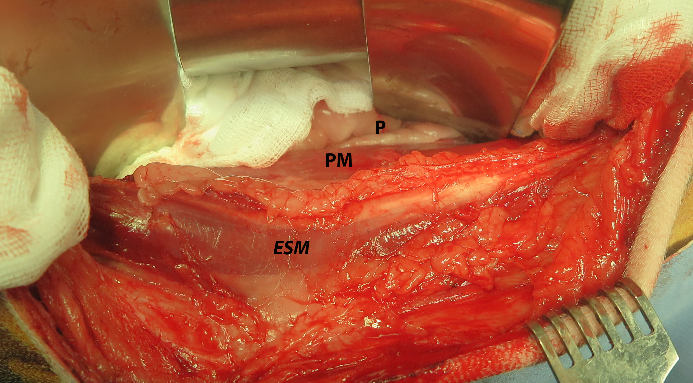


Figure 3: Photograph retraction of the peritoneal fat tissue (P) to expose the psoas muscle (PM). The erector spinae muscle (ESM) is separated from the psoas muscle (P) by the transverse processes and the middle thoracolumbar fascia

- Detach the psoas muscle from the TP with a small incision and retract the psoas muscle ventrally and medially off the TP. Confirm the appropriate level with fluoroscopy.
- Place a hohmann retractor at the L2-3 disc space and extent the incision until adequate exposure is achieved, making sure to preserve the segmental vessels and spinal nerves.
- Prepare screw insertion near the proximal endplates of each vertebral level stabilized and the distal endplates of each end vertebra. Use the TP as a reference for insertion. The screws should not extend beyond the imaginary line through the ventral side of the TP. For our study a 4.35mm x 25 mm screws were used. A 3.5 mm drill was used for the holes. The screws were intentionally placed bicortical.
- Insertion of the screws adjacent to the treatment level should always take place after cage implantation, because the screw head (tulip) may complicate cage insertion.
- Incise the annulus and use a combination of sharp spoons, curettes and rongeurs to remove most of the disc. Clean the endplates of the remaining disc tissue with a Cobb elevator preserving the subchondral bone. (Fig 4)


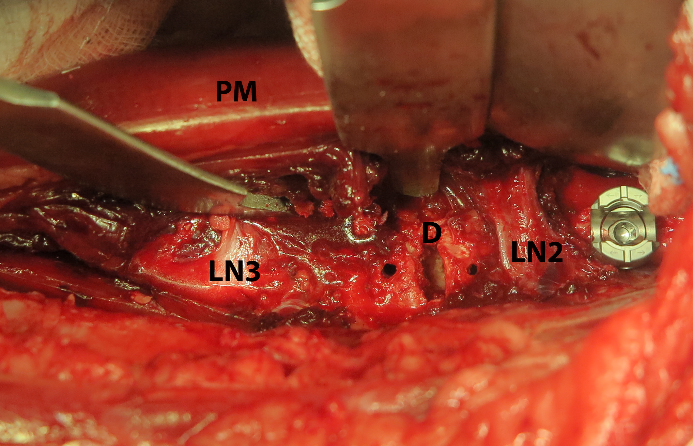


Figure 4: photograph after preparation of the L2-3 disc space(D) and for the adjacent screws (Psoas muscle (PM), 2nd and 3nd lumbar nerve (LN2 and LN3))

- Hammer the cage into place and verify radiographically and by direct vision that it does not enter the canal. The cage should be stable enough to resist being extruded by gentle manual retraction.
- Both screw and cage implantation can be difficult by the presence of nerve branches at the L4-5 level. Partially release the nerves from the underlying tissue and push gently aside with for instance a Steinmann pin.


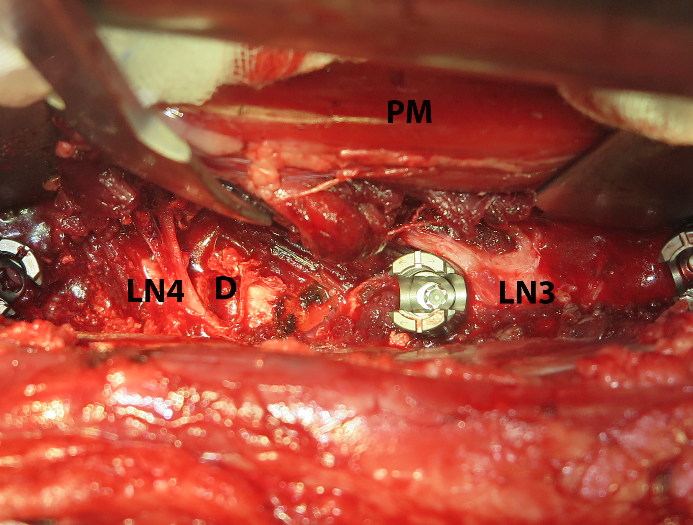


Figure 5: photograph after preparation of the L4-5 disc space(D) and for the adjacent screws. (Psoas muscle (PM), 3th and 4th lumbar nerve (LN3 and LN4))

- Contour the rod to the natural contour of the lumbar kyphosis.
- Insert the rod under direct visualisation. (Fig 6)


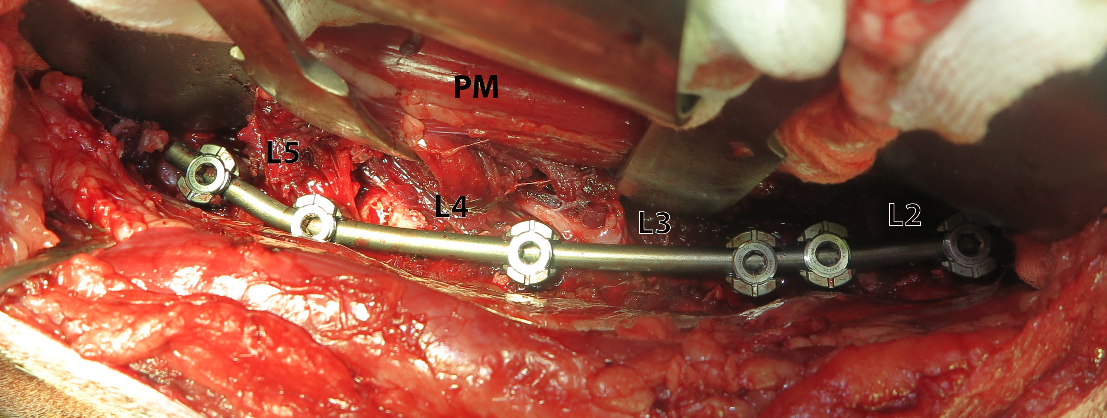


Figure 6: Photograph of the rod contoured to natural kyphosis and fixed with setscrews

- Irrigate the wound with saline and close in layers. We used resorbable sutures for the skin.
